# EVALUATION OF A LATINO BRAIN HEALTH FAIR FOR ALZHEIMER’S DISEASE EDUCATION AND RESEARCH RECRUITMENT

**DOI:** 10.1093/geroni/igae098.2588

**Published:** 2024-12-31

**Authors:** Theresa Kehne, Janet Rojina, Linda Ko, Sonia Bishop, Kelley Pascoe, Kimiko Domoto-Reilly, Israel Anaya-Carmona, Karen Torres

**Affiliations:** University of Washington, Seattle, Washington, United States; University of Washington, Seattle, Washington, United States; University of Washington, Seattle, Washington, United States; University of Washington, Seattle, Washington, United States; University of Washington, Seattle, Washington, United States; University of Washington, Seattle, Washington, United States; University of Washington, Seattle, Washington, United States; University of Washington Harborview Medical Center, Seattle, Washington, United States

## Abstract

Hispanic/Latino individuals are at higher risk of developing Alzheimer’s disease (AD) than non-Hispanic White individuals, yet risk factors in the Hispanic/Latino community are not well understood due to barriers impacting research participation. This study, in partnership with a community advisory board, developed and evaluated a novel “Brain Health Fair” (BHF) intervention for increasing AD knowledge and willingness to participate in AD research among Hispanic/Latino individuals. The BHF involved an educational presentation and interactive stations run by language-concordant health professionals that provided information about AD, health promotion and the importance of research participation to improve health equity in a culturally grounded context. Participant knowledge, attitudes, and willingness to participate in AD research were assessed in an anonymous assessment administered pre and post intervention. Participants were also given the option to enroll in the local University of Washington Alzheimer’s Disease Research Center (ADRC). Participants (n=26) demonstrated a significant change in knowledge of AD in the Latino community pre and post intervention (M pre; post=1.4;3.6, SD pre; post=1.5;1.5, Z=-3.5, p=0.001), preventative behaviors for AD (M=1;3.3, SD=1.4;1.7, Z=-3.6, p=0), what ADRC participation entails (M=0.9;3.5, SD=1.6;1.7, Z=-3.8, p=0) and benefits of research participation (M=1.2;3.9, SD=1.8;1.6, Z=-3.5, p=0.001) (two-sided Wilcoxon signed-rank test, p=0.001). After the intervention, 85% reported being satisfied and found it culturally appropriate and 81% signed consent forms to enroll in the ADRC. The BHF showed great promise for improving knowledge of AD and readiness of Hispanic/Latino individuals to participate in research and was a successful method for recruiting participants into AD research.

